# Supplementary material for: “Feeding the baby breast milk shouldn’t be a problem” breastfeeding confidence and intention in pregnant persons with type 2 diabetes mellitus from Thailand
Source: PLOS Glob Public Health. 2025 Feb 14;5(2):e0004205. doi: 10.1371/journal.pgph.0004205 (PMC11828413; doi:10.1371/journal.pgph.0004205)
Supplement: S1 File — (DOCX) [file pgph.0004205.s001.docx]

**Supplement File 1****: interview guide for pregnancy appointment**

| **Interview Questions (*Probing Questions in Italic*)** |
| --- |
| **Aim: Breastfeeding confidence and intention in pregnancy** |
| 1. What does breastfeeding mean to you? |
| 2. Describe for me, if you can, any influence having diabetes might have on your plan to breastfeed your baby. **If you have had prior breastfeeding experiences, how were they?* |
| 3. Share with me, if you can, how you came to the decision to breastfeed your baby. *Are there any factors that make you want to feed your baby breast milk? *If you have had prior breastfeeding experiences, how were they?* |
| 4. Is there anything else you would like to share or any other questions you may have? Or Is there anything you may not have thought about related to the questions from the interview? I look forward to talking with you next time. |

*Note. N/A= not applicable; * = probing questions for multigravida pregnant women*

**Thank you for your time and thoughtful responses.**
