# Supplementary material for: “Feeding the baby breast milk shouldn’t be a problem” breastfeeding confidence and intention in pregnant persons with type 2 diabetes mellitus from Thailand
Source: PLOS Glob Public Health. 2025 Feb 14;5(2):e0004205. doi: 10.1371/journal.pgph.0004205 (PMC11828413; doi:10.1371/journal.pgph.0004205)
Supplement: S2 File — (DOCX) [file pgph.0004205.s002.docx]

**Supplement File 2 Member checking**

A participant shared that*“I think this is authentic. One thing I want to add is that the breastfeeding equipment is not as expensive as I expected. For example, breast milk pumps range from approximately 200 Baht ($7) for good quality ones up to 2000 Baht ($60) or maybe more. It depends on a mother’s financial status and how much she has to spend on this equipment. I think the main factor for breastfeeding is whether the mom really wants to breastfeed a baby or not…that is the main factor and other factors may depend on the mother’s status such as her job, income, or financial status.”*

This member-checking process was instrumental in enhancing the credibility of our qualitative findings.
