# Supplementary material for: “Feeding the baby breast milk shouldn’t be a problem” breastfeeding confidence and intention in pregnant persons with type 2 diabetes mellitus from Thailand
Source: PLOS Glob Public Health. 2025 Feb 14;5(2):e0004205. doi: 10.1371/journal.pgph.0004205 (PMC11828413; doi:10.1371/journal.pgph.0004205)
Supplement: S1 Data — (DOCX) [file pgph.0004205.s004.docx]

**Supplement File 6 Dataset**

| **ID** | **Status** | **Age (year)** | **Marital status** | **Education** | **Family income (Baht)** | **Occupation** | **Para** | **Gestation (week)** | **T-IFI**  **score** | **BSES-SF**  **score** |
| --- | --- | --- | --- | --- | --- | --- | --- | --- | --- | --- |
| #001 | Enrolled | 37 | marriage | bachelor's degree | 50,000 | government | Not given birth | 37 | 14.00 | 55.00 |
| #003 | Enrolled | 38 | marriage | higher than bachelor’s degree | 70,000 | government | Given birth | 14 | 16.00 | 53.00 |
| #004 | Enrolled | 35 | marriage | diploma | 34,000 | private company | Given birth | 38 | 12.00 | 33.00 |
| #005 | Enrolled | 27 | marriage | secondary school | 30,000 | merchant | Given birth | 7 | 15.00 | 17.00 |
| #006 | Enrolled | 40 | marriage | bachelor's degree | 30,000 | private company | Given birth | 36 | 16.00 | 51.00 |
| #007 | Enrolled | 32 | marriage | higher than bachelor’s degree | 50,000 | government | Not given birth | 36 | 10.50 | 40.00 |
| #009 | Enrolled | 40 | marriage | diploma | 65,000 | government | Not given birth | 16 | 2.00 | 59.00 |
| #010 | Enrolled | 28 | marriage | secondary school | 30,000 | freelance | Given birth | 7 | 5.00 | 52.00 |
| #011 | Enrolled | 34 | marriage | certification | 50,000 | government | Not given birth | 9 | 1.50 | 50.00 |
| #013 | Enrolled | 32 | marriage | higher than bachelor’s degree | 40,000 | agriculture | Given birth | 13 | 16.00 | 59.00 |
| #014 | Enrolled | 37 | marriage | secondary school | n/a | freelance | Given birth | 12 | 12.50 | 28.00 |
| #015 | Enrolled | 32 | marriage | bachelor's degree | 10,000 | unemployed | Given birth | 32 | 4.50 | 43.00 |
