# Supplementary material for: “Feeding the baby breast milk shouldn’t be a problem” breastfeeding confidence and intention in pregnant persons with type 2 diabetes mellitus from Thailand
Source: PLOS Glob Public Health. 2025 Feb 14;5(2):e0004205. doi: 10.1371/journal.pgph.0004205 (PMC11828413; doi:10.1371/journal.pgph.0004205)
Supplement: Attachment — Submitted filename: PGPH Response to reviewers R1 (10-18-2024).docx [file pgph.0004205.s007.docx]

**PGPH-D-24-01048**
"Feeding the baby breast milk shouldn’t be a problem" Breastfeeding confidence and intention in pregnant persons with type 2 diabetes mellitus from Thailand

Dear Editor and Reviewers,

We appreciate the opportunity to submit our revised manuscript. Thank you for dedicating your time and effort to providing valuable feedback on our work.

We have addressed the reviewers' comments and concerns in a point-by-point manner. Please find our responses to each of them outlined below. We believe the manuscript has been considerably strengthened with these revisions.

| **Reviewer #1 comments** | **Authors’ Response** |
| --- | --- |
| This study described breastfeeding confidence and intention in Thai women with T2DM. The study employed semi-structured interviews, quantitative data collection, and directed content analysis using the NIMHD as a guiding framework to develop codes and themes. The findings on breastfeeding intentions, confidence, barriers and facilitators at individual, interpersonal and societal levels are valuable contributions to our understanding of breastfeeding confidence and intention in Thai pregnant persons with T2DM. | Thank you for your insightful suggestions. |
| The strengths of this study include: 1. The incorporation of an appropriate theoretical framework to guide analysis and the examination of themes across different levels of influence 2. The establishment of rigor/trustworthiness of findings by member-checking and other methods 3. The concurrent presentation of both quantitative and qualitative data | Thank you for your positive feedback! |
| I recommend several areas for major revision to improve the manuscript as a whole. These suggested revisions are mostly related to providing additional detail in the Methods and Results section, improving clarity of language, providing additional literature support in the Discussion section, among other items. Please also include continuous line numbers in the manuscript file upon resubmission. | Thank you for your suggestion. Please see a revised manuscript for your review. |
| 1. The NIMHD framework is an important part of the study design/analysis, yet its description in the Methods section needs more detail. What was the rationale for using this framework? How did you employ this framework (e.g., a reader can figure out later on that you used the framework to divide themes, so perhaps you can state that in the Methods section)? | We revised as suggested. See Page 6, Lines 149-157.  “The NIMHD framework was chosen because it considers health disparities across multiple domains (e.g., biological, behavioral, sociocultural) and levels (i.e., individual, interpersonal, community, and societal) [18]. The framework aligns well with Thai culture, where the majority of people traditionally live in extended family units. This cultural fit makes it particularly relevant for understanding health and social dynamics within Thai society. Pregnant persons in Thai culture collaborate, learn, and share health practices, including diabetes management and breastfeeding, with other generations, including grandparents and relatives [19, 20]. Theoretical framework integration spanned across the study design, data collection, data analysis procedures, and results presentation.” |
| 2. It is not clear how the authors decided on the question to focus on for member checking, or why only one participant was selected— were other participants asked to partake in the member-checking? Some additional detail on this in the Methods section would be valuable. | We added information as suggested. See Page 10, 253-257.  “We prepared a diagram with visual summaries, which allowed both a researcher and one participant (10 percent of enrolled participants) to review the relationships between themes and subthemes after all data collection was completed [31]. We selected one participant who had recently given birth to review the results, as she could best recall her pregnancy experience.” |
| 3. The short descriptions of themes (in the Results/Themes section) need additional detail (not more than 1-2 sentences each, though) to clarify what the themes are illustrating/what participants expressed in relation to the theme. For example, under theme 4 (Breastfeeding Facilitators”), I suggest rephrasing the sentence to “Facilitators to breastfeeding included considerations of cost, family support and resource availability” or something similar so the reader understands the overarching message of the theme. | Thank you for your suggestions. Please see revision under the result section. Pages 14-19. |
| 4. The inclusion of dietary considerations/eating of traditional Thai foods under the Interpersonal level of the Breastfeeding Facilitators theme seemed underexplained to me. What is the rationale for considering this as Interpersonal? Is it because food beliefs are considered peer/family norms? It may be helpful to return to the NIMHD framework for this section. | We added more information on Page 18, Lines 424-425. We linked traditional foods, passed down from one generation to the next, as part of family norms, which fall under the interpersonal level of the NIMHD framework.  “In addition, several participants (4/12) in the study shared their beliefs that consuming traditional Thai foods and herbs such as Mai Nom Nang, ginger, and banana blossom in addition to prescribed medication can increase breast milk supply. These beliefs were passed down from generation to generation as family norms. For example, one participant's grandmother made her an herbal juice called Mai Nom Nang during her maternity leave, they also consumed banana blossom as well. Another participant consumed typical Thai foods that were recommended for breastfeeding mothers, such as ginger and banana blossom, and did not take any medication to boost her milk supply (ID003).” |
| 5. Are there any additional details about the study setting that can be included? | We provided information about study setting. Please see Pages 6-7, Lines 162-177.  “This study recruited pregnant persons with T2DM at a medical tertiary hospital in Bangkok, Thailand. As of 2023, Bangkok has an estimated population of approximately 10.5 million people. A recent national survey in Thailand reported the diabetes prevalence rate increased from 8.3% in 2004 to 10.8% in 2014 [21].  Thailand has experienced rapid social and economic changes, leading women to enter the workforce in greater numbers, from 44% in 1980 to 59.2% in 2019 [22]. Mothers who return to work are more likely to discontinue breastfeeding, and 31% of mothers send their infants to live with their grandmothers in other areas as they go back to work in Bangkok [22].  Our data collection site is a designated Baby-Friendly Hospital and has breastfeeding clinics where lactation support providers offer ongoing support to persons who breastfeed or encounter difficulties in breastfeeding. The hospital also promotes immediate skin-to-skin contact, encourages breastfeeding on demand, and assists mothers in initiating breastfeeding within the first hour after birth.” |
| 6. The use of gender-neutral pronouns is not consistent throughout. Thether the authors decide to use gender-neutral pronouns or pronouns based on participants’ identified gender (if collected during the study) is up to them, as long as it is consistent. I recommend the same for the term “pregnant persons” — this is used in the manuscript, but the term “women” is used in the abstract. | Thank you for pointing this out. We have decided to use the gender-neutral term 'pregnant persons' and have consistently used it throughout our manuscript. |
| 7. The Discussion section would benefit from additional literature to support the study’s findings and place them in dialogue with existing knowledge. For example, the sentence stating “previous literature found pregnant persons with T2DM were more likely to not exclusively breastfeed [33]” would benefit from additional detail about that study’s setting, the results and other literature supporting this finding. | We revised discussion section as suggested. See Pages 19-22. |
| 8. Under Theme 2, it is stated that participants were worried about the effect of T2DM on milk, but then it is stated that all but one participant expressed their confidence in breastfeeding. This seems like an inconsistency between the conclusion from the data and the actual findings. | We clarified this point and revised Theme 2. See Pages 15-16.  **“Individual level.** Participants (11/12) expressed their confidence in breastfeeding, because they believed that diabetes has no effect on breastfeeding if they can control their blood sugar levels, *“I don’t think it[diabetes] has any effect. This is my opinion. I haven’t consulted the doctor about this...I think that if I eat according to the doctor’s recommendations, I will be able to manage the blood sugar level during the breastfeeding period”* (ID004). However, one participant (1/12) expressed worry and uncertainty regarding whether having diabetes would negatively affect the quality of breast milk, *“I’m worried about whether there will be any effects on my baby if it is fed breast milk…I’m worried about the scenario that, when the time [breastfeeding] comes, I may not be able to take care of myself as well as I do now. My blood sugar level may not be stable due to hormones or something like that. I’m worried about whether there would be any effects on my baby if it were fed breast milk* (ID009).” |
| 9. Tables should be labelled as Table S1, S2, etc since they are all supplementary files. Captions should be formatted as "Table S1 Text. _______". Please double-check the PLOS Global Public Health guidelines for all tables. | Thanks for pointing that out. We have added tables and embedded them in the manuscript text. |
| 10. In the Discussion, page 17, the phrase “This cultural awareness” in regards to healthcare providers offering information on diet’s impact on breast milk seems a little awkward to me. Is “cultural” the right word? Perhaps reconsider, and use a term like “improved awareness.” | We revised on Page 21, Lines 543. “This improved awareness helps support breastfeeding goals and ensures the well-being of both mother and child.” |
| 11. There are some places where sentences are not clear (possibly due to typos) or are in need of transitions/relocating— I would be sure to check for typos throughout. Here are the examples I found, by page number: | Thank you for pointing this out. We have checked for typos throughout and addressed them accordingly. |
| 1. Page 4: “were found to be significantly positive correlations” — there is a word missing here | We revised to “were positively correlated” See Page 4, Line 92. |
| 2. Page 6: Move the sentence about the gift cards to the paragraph above. | We moved as suggested. See Page 7, Lines 186-187.  “Participants were compensated with a $10 gift card (equivalent to 300 Baht) upon completion of data collection.” |
| 3. Page 11: 1. “On Google. I’ve just studied…” — do you mean, “On Google, I’ve just studied…”? | We revised on Page 16, Line 355.  *“On Google, I’ve just studied whether it would be safe for a person with diabetes to become pregnant”* (ID011). |
| 2. “We did not find interpersonal barriers.” Perhaps you could rephrase this as “We did not find that participants reported breastfeeding barriers at the interpersonal level” | We revised on Page 16, Lines 373-374.  “We did not find participants reporting any breastfeeding barriers at the interpersonal level.” |
| 4. Page 13 — “and they also consumed banana blossom” — please change to “they consumed banana blossom as well.” | We revised on Page 18, Lines 432.  “they also consumed banana blossom as well.” |
| 5. Page 15: “Longitudinal research measuring maternal intention during breastfeeding and after childbirth” is an incomplete sentence. | We revised on Page 22, Lines 569-571.  “We suggest that future longitudinal research measures maternal intention and confidence before their first birth, during breastfeeding, and before their next birth.” |
| 6. Page 16: Participants did not share that breastfeeding shipping costs as a barrier” — there seems to be a word missing here. Thank you for the opportunity to review this important work. | We revised on Page 22, Lines 578-647.  “In addition, participants recognized breast milk’s benefits and cost-effectiveness, with breastfeeding shipping services helping maintain breastfeeding despite separation. Integrating milk shipping into prenatal education could reduce reliance on formula and support family planning. [22].” |

| **Reviewer #2 comments** | **Authors’ Response** |
| --- | --- |
| The paper shows a lot of promise and is very interesting! My biggest concern is the lack of emphasis on T2DM throughout the paper. More needs to be added into the paper in all areas (background, results, and discussion) on the experience of T2DM particularly, so that the paper focuses on how having diabetes impacts this sample’s intention to breastfeed and confidence in their ability to breastfeed in the future. I think it would be especially interesting to focus more on how many of the participants did not discuss it with their providers despite having concerns. It was mentioned briefly in your discussion but not in your findings. More details on this – and other examples of barriers specific to T2DM rather than general pregnancy/motherhood – would enrich your paper. | Thank you very much for your suggestions. We have added more sections and citations related to T2DM in the background, results, and discussion. Please find the revised manuscript for your review. |
| Title - The non-quotation portion of your title should include the topic of breastfeeding that ties into your paper. At the moment it is very ambiguous and does not focus on the main issues that your paper addresses. For example: “Breastfeeding plans/intentions of pregnant women…” | Revised the title to "Feeding the baby breast milk shouldn’t be a problem" Breastfeeding confidence and intention in pregnant persons with type 2 diabetes mellitus from Thailand |
| Abstract - The fourth sentence needs restructuring for ease of reading. For example: “This qualitative analysis utilized data from a parent study with…” | We revised on Page 3, Line 53.  This qualitative analysis utilized data from a parent study with a convergent parallel mixed-methods design. This study was guided by the National Institute on Minority Health and Health Disparities (NIMHD) Framework. |
| - The final sentence needs a comma between ‘confidence’ and ‘barriers’, rather than an ‘and’ | We revised as suggested. |
| Introduction Overall: - More information on Thailand would be useful to contextualize: pregnancy care, rate of T2DM in the population, how T2DM is cared for in the population, current breastfeeding rates (eg. initiation, EBF rates, any BF rates, how long they typically BF for), and why increasing breastfeeding rates would be beneficial. | We added more info from Thai statistics.  Page 4, Lines 87-89 “Thailand has low breastfeeding rates. In 2019, 34% of infants were breastfed within the first hour after birth; only 14% were exclusively breastfed during the first six months, down from 23% in 2016 [4].”  Page 5, Lines 118-123 “A recent study of Thai persons younger than 30 years of age, showed that the average age at diagnosis is 20.8 years old. The prevalence of diabetes at younger ages (<30 years) is greater in females (60.2%) than in males (39.8%) [13]. The increasing prevalence of diabetes among younger women is particularly important as they contemplate and plan for pregnancy. The number of pregnant women with preexisting diabetes, rarely seen 30 years ago, has more than doubled from 0.7% to 1.5% of all pregnancies [14].” |
| - Why is this question important? You need to include more on why knowing the answer to how T2DM impacts breastfeeding initiation in Thailand is of interest to the reader. | Revised on Page 5, Lines 125-128.  “Understanding how T2DM impacts breastfeeding initiation in Thailand is important because it can inform targeted interventions to improve breastfeeding rates. This knowledge is crucial for improving maternal and infant health outcomes in a country where breastfeeding rates are already suboptimal.” |
| First Paragraph: - Remove the word ‘universally’ from your first sentence to help with flow of reading. | We revised as suggested. |
| - You discuss EBF in the first 6 months. Does this mean ANY duration of time EBF or (what I think you are referencing) still EBF by 6 months? There is a big difference between initiation of EBF and then continuation up to 6 months in the statistics – especially in reference to your 2nd, 4th, and 5th sentences in that first paragraph – so be sure to clarify more specifically what those numbers refer to. | We added more info in the introduction.  Page 4, Lines 87-89 “Thailand has low breastfeeding rates. In 2019, 34% of infants were breastfed within the first hour after birth; only 14% were exclusively breastfed during the first six months, down from 23% in 2016 [4].” |
| Second Paragraph: - In the second sentence, it should either be “found to be significantly correlated…” or “found to have…” | We revised as suggested. |
| Third Paragraph: - Define briefly the three types of diabetes that you mention. Then give a more in-depth explanation of T2DM as this is the one that you will focus on in your paper. | We revised on Pages 4-5, Lines 99-116.  Diabetes is a common complication during pregnancy. Pre-gestational diabetes mellitus (PGDM) includes women with type 1 diabetes (T1DM: insulin deficiency) and type 2 diabetes (T2DM: insulin resistance) [10]. Gestational diabetes mellitus (GDM) is hyperglycemia during pregnancy [10]. High blood sugar levels delayed onset of lactation and negatively impact breastfeeding plans [11]. Persons with PGDM are more likely to stop breastfeeding compared to those without diabetes before pregnancy [12]. A recent study [9] found that breastfeeding initiation rates were lower in persons with T2DM compared to those with T1DM, with predictors of initiation failure in T2DM including the intention to partially breastfeed. |
| - Why does having T2DM impact breastfeeding initiation? Add some more details on this. | We added the rationale on Pages 4-5.  High blood sugar levels delayed onset of lactation and negatively impact breastfeeding plans [11]. |
| Material and Methods Overall: - Instead of saying the position (eg. PI, non-Thai researcher, etc), put the initials of the person in brackets next to their role. | We revised the document as suggested by adding our team members' initials. |
| Research Design: - Same comment as in the abstract that the first paragraph can be rephrased to help ease of reading. | We revised on Page 5, Line 132.  “This qualitative analysis utilized data from a parent study with a convergent parallel mixed-methods design [15].” |
| - If you used both qualitative and quantitative then wouldn’t your study be mixed-methods instead? Especially if you used the T-IFI and BSES-SF which both have Likert scales for outcomes. | We utilized data from a parent study with a convergent parallel mixed-methods design. Yes, we used the T-IFI and BSES-SF, which corresponded to themes 1 and 2. However, we did not have quantitative measures to combine with themes 3 and 4, which addressed breastfeeding barriers and facilitators. |
| Setting and Relevant Context: - More information would be useful here. What is the population in Bangkok? What does it mean to be designated Baby-Friendly for breastfeeding rates/outcomes (eg. encourages skin-to-skin, no formula advertised, mandatory BF training, etc)? | Revised on Pages 6-7, Lines 163-178.  “This study recruited pregnant persons with T2DM at a medical tertiary hospital in Bangkok, Thailand. As of 2023, Bangkok has an estimated population of approximately 10.5 million people. A recent national survey in Thailand reported the diabetes prevalence rate increased from 8.3% in 2004 to 10.8% in 2014 [21].  Thailand has experienced rapid social and economic changes, leading women to enter the workforce in greater numbers, from 44% in 1980 to 59.2% in 2019 [22]. Mothers who return to work are more likely to discontinue breastfeeding, and 31% of mothers send their infants to live with their grandmothers in other areas as they go back to work in Bangkok [22].  Our data collection site is a designated Baby-Friendly Hospital and has breastfeeding clinics where lactation support providers offer ongoing support to persons who breastfeed or encounter difficulties in breastfeeding. The hospital also promotes immediate skin-to-skin contact, encourages breastfeeding on demand, and assists mothers in initiating breastfeeding within the first hour after birth.” |
| Sample: - Define what purposive sampling means. | Revised on Page 7 Lines 180-182.  “Participants were recruited from the antenatal care clinic through purposive sampling, which targets information-rich cases that best contribute to the research questions based on eligibility criteria [23].” |
| - In the second sentence, remove the word “comprised”. | Revised as suggested. |
| Measurement: - Second Paragraph: The last sentence does not make sense, change “human milk” to “nutrition”. | Revised as suggested on Page 8, Line 207. |
| Data Collection: - Explain what directed content analysis entails. | We added definition on Page 9, Lines 239-241.  “Data analysis was based on directed content analysis where researchers conducted analysis with the predetermined codes derived from the NIMHD framework [29].” |
| - When you say that a researcher and a participant review the relationships between themes and subthemes, do you mean that you checked along the way with differing participants or that you used one in particular? Clarify this. If only one participant was used, how did you pick which one? Did you discuss during the data collection or after? Etc. | We clarified how we conduct member checking on Page 9, Lines 254-258.  We prepared a diagram with visual summaries, which allowed both a researcher and one participant (10 percent of enrolled participants) to review the relationships between themes and subthemes after all data collection was completed [31]. We selected one participant who had recently given birth to review the results, as she could best recall her pregnancy experience. |
| Results Overview: - More detail is needed in text for your results. Although it is in your tables it is helpful to the reader if the important values are also in the text. | Thank you. The details of the themes and subthemes were added. As an example:  **Theme 1: Breastfeeding intention in pregnancy**  This theme identified three levels of influence (i.e., individual, interpersonal, and societal), with most findings falling within the individual level. Participants planned to breastfeed until their milk supply ran out, aiming for 3 months to 1 year, or until the baby stopped nursing. At the interpersonal level, participants planned to breastfeed until their baby stopped actively nursing. At the societal level, their intention to breastfeed was also influenced by maternity leave. (Page 14, Lines 305-310). |
| Sample Characteristics: - Add in more data for the stats given. What was the SD for the age and monthly income? What is the percentage of the sample educated higher than secondary school, working in government or state, primipara and multipara? Include mean gestation. | We added more information on Pages 10, Line 271-284.  “Twelve Thai pregnant individuals with T2DM, mean age 34.33 (Standard Deviation: SD = 4.29) years, participated. All participants were married, with 25% holding a bachelor’s degree and another 25% holding a degree higher than a bachelor's. 41.7% worked in government or state enterprises. The mean monthly household income was 41,727.27 Baht (SD = 17,401.67). Gestational age ranged from 7 to 38 weeks (Mean = 21.42, SD = 13.03). Participants were diagnosed with T2DM between 3 weeks and 10 years prior (Mean = 45.42 months, SD = 41.68). Of 12 participants, seven previously gave birth (primipara/multipara), while five were pregnant for the first time (nullipara). See Table 1 for demographic details.” |
| - You need to specify that the diagnosis is for T2DM | We revised on Page 11, Line 282. |
| - Give the SD and range for T-IFI score and BSES-SF | We added more information on Page 12, Lines 290-294.  In Table 2, the T-IFI mean score was 10.41 (SD = 5.63), indicating that they have a higher intention to breastfeed the baby. The mean BSES-SF score was 45 (SD = 13.18). The participants were divided into two groups based on their BSES-SF total scores, with the mean score for the low-confidence group being 33.50 (SD = 11.94) and the mean score for the high-confidence group being 54.85 (SD = 3.00) (Table 2). |
| - How did you divide the participants into two groups for the BSES-SF, clarify. Eg. “Low-confidence group (<50) being… high-confidence group (>50) being…” | According to Nanishi et al. 2015, scores equal to or less than 50 were indicative of low confidence in breastfeeding, while scores above 50 indicated high confidence in breastfeeding. We have this information on Page ,8 Line 212-214. |
| Qualitative Findings Overview: - Include numbers next to all your claims. For example: “Some participants (3/12) indicted that…” | We added number of participants each theme. Please see a revision from Pages 14-19. |
| - If only one participant expressed concern over their ability to feed their baby should that quote be used as your title? The title sets it up as if this is a dominant theme that was found. It also implies a sense of negativity, whereas the interview themes seem very positive. Instead, it may be better to use another quote from a theme that was more prominent such as the intention to breastfeed until they stop producing milk (if this is indeed a large percentage). | Revised the manuscript title to represent the overall finding from our study.  Our revised title "Feeding the baby breast milk shouldn’t be a problem" Breastfeeding confidence and intention in pregnant persons with type 2 diabetes mellitus from Thailand |
| Theme 3: - (Individual) This paragraph was difficult to read, rephrase if possible. Additionally, in the last sentence there is an error “that wasn’t was not enough”. | Thanks for pointing this out. We revised this paragraph as follows (Page 17 Lines 375-379):  **Individual level.** A common individual-level barrier reported by participants (2/12) was a difficult experience in breastfeeding with a previous child, including issues like insufficient milk supply and problems with the baby's latch. For example, one participant (ID014) wanted to breastfeed a baby for six months but could not do it for any of them. Sometimes, they could only pump a small amount of breast milk, which was not enough for the baby (ID014). |
| - (Societal) I am not sure if this would count as a societal level issue and not a personal? Unless it is expected of them by society to send away their infants? If this is a common societal practice/expected of mothers, please explain more in the background to help contextualize the reader. | We added the rationale for how we coded this at the societal level as follows (Page 17, Lines 380-386):  **Societal level.** In Thai society, it is common for mothers to send their infants to be cared for by relatives in different provinces due to work or family obligations. This physical distance between participants (4/12) and the baby was reported as a significant barrier to breastfeeding, as it made it difficult for mothers to provide breast milk regularly. Participants had planned to send their babies to be cared for by family members after their three-month maternity leave ended. Two participants shared *“Because I’ll send the baby to another province”* (ID001) and *“I send a baby to be cared for by grandma”* (ID006). |
| - (Societal) Include how many of the mothers were planning on living in different provinces from their infants. | We added numbers of participants who have planed on living in different provinces from their infants.  (Page 17, Lines 380-383):  **Societal level.** In Thai society, it is common for mothers to send their infants to be cared for by relatives in different provinces due to work or family obligations. This physical distance between participants (4/12) and the baby was reported as a significant barrier to breastfeeding, as it made it difficult for mothers to provide breast milk regularly. |
| Theme 4: - You say that each level has two subthemes but I am unable to identify what they are. Make them more explicit. | We clarified this point in Table 3, describing themes and subthemes, as well as providing detailed descriptions of the subthemes (Page 17, Lines 388-392).  The theme of breastfeeding facilitators identified three levels of influence: individual, interpersonal, and societal levels. The individual facilitators of breastfeeding included considerations of cost; the interpersonal facilitators were the significance of bonding, family support, the consumption of Thai food and herbs; the societal facilitator was the availability of resources. |
| - (Individual) At the end you have an ID but there is no direct quote. | We added a quotation on Page 18, Line 420-421.  One said, *“There’s a greater bond that comes from feeding the baby breast milk, especially if the baby suckles from my breast”* (ID013). |
| - (Individual) How are the benefits and bonding aspect of breastfeeding a facilitator? I think they should be their own thing separated from cost-effectiveness and immune boosting – which it is clear how those two are classed as facilitators. | Yes we have two subthemes under individual facilitators 1) breast milk is beneficial and  cost-effective and 2) breastfeeding signifies a bond.  We revised on Pages 17-18, Lines 412-421 as follows:  **Individual level.** Two subthemes under individual facilitators 1) breast milk is beneficial and cost-effective and 2) breastfeeding signifies a bond. First, most participants (7/12) were aware of breastfeeding benefits and cost-effectiveness compared to infant formula. One shared that *“Breast milk is beneficial*. *Breast milk is more cost-effective than infant formula. Because I would only pay for the shipping”* (ID006). A participant stated that breastfeeding strengthened a baby’s immune system: *“the longer the baby is fed breast milk, the stronger it will be”* (ID015). Second, many of the participants (5/12) shared that breastfeeding signifies a bond between mother and baby. When the participants breastfed the child, they felt the connection, which made them more willing to breastfeed. One said, *“There’s a greater bond that comes from feeding the baby breast milk, especially if the baby suckles from my breast”* (ID013). |
| - (Interpersonal level) in the final sentence it should either be “they planned to restrict their diet…” or “she planned to restrict her diet…” | We revised to “Therefore, they planned to restrict their diet more during breastfeeding.” (Page 19, Line 453) |
| - (Societal level) There are a lot of typos in this section. | We revised the paragraph (Page 19, Lines 454-460):  **Societal level.** Participants (5/12) found it beneficial to utilize breastfeeding equipment, such as a breast pump and a freezer, to facilitate breastfeeding. One participant used a breast pump after returning to work, bringing it to pump milk at the workplace and then taking the milk home (ID013). A breast milk shipping service, estimated at 200-300 Baht per delivery, involved storing and transporting expressed milk from the mother’s home to where the baby was staying. Participants preferred door-to-door services, like 'Inter Express,' over coach services, which required their relatives to pick up the expressed milk at a coach station (ID006). |
| - (Societal level) What do you mean by “alternatively” in this paragraph? Alternative to what? | We revised this sentence and removed “Alternative” |
| Member Checking: - This section has a lot of typos and grammatical mistakes. | We revised this section (Page 19, Lines 462-467):  The PI (PR) conducted a phone meeting with an enrolled participant for member checking. The participant agreed with the finding. The participant added *“It depends on a mother’s financial status and how much she has to spend on this equipment. I think the main factor for breastfeeding is whether the mom really wants to breastfeed a baby or not…that is the main factor and other factors may depend on the mother’s status such as her job, income, or financial status”* (see Supplement File 2). |
| - The final sentence does not make sense. How do you draw the conclusion that as affordability depends on the mother’s financial status that the key factor is mother’s desire? Surely this sentence shows that the key factor is income/job/financial status and not her desire? | We added a participant’s quotation. We revised this section (Page 19, Lines 463-467):  The participant added “It depends on a mother’s financial status and how much she has to spend on this equipment. I think the main factor for breastfeeding is whether the mom really wants to breastfeed a baby or not…that is the main factor and other factors may depend on the mother’s status such as her job, income, or financial status” (see Supplement File 2). |
| Discussion Overview - Not much emphasis or discussion on the impact of T2DM particularly in your findings – you do not mention any T2DM findings until paragraph 4. You need to focus more on how T2DM interplays with the pregnant women’s breastfeeding intentions and confidence. Focus on T2DM first in your discussion as this should be the main focus of your paper. You can then move to more general recommendations later (eg, the need to extend maternity leave for all mothers). | We revised the discussion section and added more citations related to breastfeeding and diabetes to deepen our responses. Please refer to Pages 19-23. |
| - The discussion is very disjointed, as each paragraph addresses a finding without leading into the next. There needs to be more flow and tying the points together to make a larger overview rather than 9 separate points. This may be assisted when integrating diabetes into each point. | We revised and reorganized the discussion section to better connect with breastfeeding and diabetes, particularly T2DM. We also referred back to the literature and included the locations and results of previous studies. See Pages 19-22. |
| Paragraph 2: - Did all participants express intention to BF until they exhausted their breast milk or just most/some? | We clarified this point by referring to it as “Some participants…" See Page 21, Line 552. |
| Paragraph 3: - You claim that these findings are similar to other studies that show that breastfeeding experience is a predictor for subsequent breastfeeding – however, as you interview women while they are pregnant you only measure breastfeeding intention and are unable to state if your participants actually breastfed or not. Be careful with phrasing in this regard. The final sentence is incomplete. | We added the suggestion for longitudinal measurement as follows:  “We suggest that future longitudinal research measures maternal intention and confidence before their first birth, during breastfeeding, and before their next birth (Page 22, Line 569-571). |
| Paragraph 4: - You discuss how participants were worried that BF would cause high blood sugar and were unsure if feeding BM would be harmful to their babies, but you do not mention this in your results so this is the first that the reader is discovering this. Add this finding into your results so you can address it better in the discussion. | We added a quotation in the results section where one participant expressed concern about high blood sugar during breastfeeding (Page 16, Lines 348-351).  “However, one participant (1/12) expressed worry and uncertainty regarding whether having diabetes would negatively affect the quality of breast milk, *“I’m worried about whether there will be any effects on my baby if it is fed breast milk…I’m worried about the scenario that, when the time [breastfeeding] comes, I may not be able to take care of myself as well as I do now. My blood sugar level may not be stable due to hormones or something like that. I’m worried about whether there would be any effects on my baby if it were fed breast milk* (ID009). |
| - This paragraph should be a bigger emphasis in your discussion – as the impact of T2DM on BF is the main driver of your paper. Expand more on this content. | We revised the discussion section to place more emphasis on the impact of T2DM on breastfeeding. |
| Paragraph 5: - Same as previous paragraph, this key finding was not discussed in your results section. Be sure to add it in. - This finding is very important! Be sure to emphasize it more. | Thank you for pointing that out. We have added a quotation from a participant who believed that,*“I don’t think it[diabetes] has any effect. This is my opinion. I haven’t consulted the doctor about this...I think that if I eat according to the doctor’s recommendations, I will be able to manage the blood sugar level during the breastfeeding period”* (ID004).  See Page 16, Lines 343-345.  We then expand upon the findings by discussing the impact of a lack of person-provider discussions on low health literacy regarding breastfeeding. See Page 20, Lines 514-535.  Participants noted that they had not discussed their breastfeeding plans with their providers including whether they could breastfeed their baby with T2DM. Our findings align with a previous nationwide survey in Japan, which explored breastfeeding support for persons with GDM and identified barriers to its promotion [36]. Participants with GDM reported that breastfeeding support was lacking during pregnancy [36]. A lack of person-provider discussion may cause low health literacy on breastfeeding. Health literacy acts as a protective factor in maintaining exclusive breastfeeding and against early cessation during postpartum, as reported by 343 participants recruited from three hospitals in Spain [37]. In Thailand, mothers commonly visit lactation clinics and attend breastfeeding counseling [8]. Hospitals could improve accessibility to specialized breastfeeding clinics, offering ongoing care from providers. Tailored counseling can enhance health literacy and address concerns of pregnancy with T2DM. |
| Paragraph 6: - This is the context we needed earlier to understand better the results on mother/infant separation. I would move the background information into the Setting and Relevant Context section. | We added information on the statistics regarding mother-and-child separation due to work in the setting and relevant contact section as follows (Pages 6-7):  Thailand has experienced rapid social and economic changes, leading women to enter the workforce in greater numbers, from 44% in 1980 to 59.2% in 2019 [22]. Mothers who return to work are more likely to discontinue breastfeeding, and 31% of mothers send their infants to live with their grandmothers in other areas as they go back to work in Bangkok [22]. |
| Paragraphs 8 & 9: - Are these foods safe/recommended for people with T2DM?  - These need to be tied into your specific sample of women with T2DM, rather than just general pregnancy as it is phrased now | We added more information on Page 21, Lines 536-544:  For the T2DM group, it’s important to collaborate with healthcare providers, like dietitians, to create a personalized meal plan that meets their health needs during breastfeeding. In this study, participants planned food restrictions, avoiding raw and fatty foods, believing it could affect breast milk composition. However, the CDC advises that breastfeeding individuals don’t need to avoid specific foods [38], and previous research has shown that maternal food restrictions are unnecessary unless the baby shows a negative reaction to the food [39]. To prevent unnecessary dietary restrictions, providers should give accurate information on how diet impacts breast milk and the baby's health. This awareness helps support breastfeeding goals and ensures the well-being of both mother and child. |

We appreciate your thoughtful comments and suggestions. We believe through the revision process; the manuscript has been improved. Thank you in advance for your consideration.

We look forward to hearing from you.

Sincerely,

Authors
